# The Alkamide *trans*-Pellitorine Targets PPARγ via TRPV1 and TRPA1 to Reduce Lipid Accumulation in Developing 3T3-L1 Adipocytes

**DOI:** 10.3389/fphar.2017.00316

**Published:** 2017-05-31

**Authors:** Barbara Lieder, Mathias Zaunschirm, Ann-Katrin Holik, Jakob P. Ley, Joachim Hans, Gerhard E. Krammer, Veronika Somoza

**Affiliations:** ^1^Christian Doppler Laboratory for Bioactive Aroma Compounds, Faculty of Chemistry, University of ViennaVienna, Austria; ^2^Department for Nutritional and Physiological Chemistry, Faculty of Chemistry, University of ViennaVienna, Austria; ^3^Symrise AGHolzminden, Germany

**Keywords:** adipogenesis, miRNA, fatty acid synthase, *Piper nigrum*, fatty acid uptake

## Abstract

Adipose tissue is an important endocrine organ in the human body. However, pathological overgrowth is associated with chronic illness. Regulation of adipogenesis and maturation of adipocytes via bioactive compounds in our daily diet has been in focus of research in the past years and showed promising results for agonists of the ion channels transient receptor potential channel (TRP) V1 and A1. Here, we investigated the anti-adipogenic potential and underlying mechanisms of the alkamide *trans-*pellitorine present in *Piper nigrum* via TRPV1 and TRPA1 in 3T3-L1 cells. *trans-*pellitorine was found to suppress mean lipid accumulation, when applied during differentiation and maturation, but also during maturation phase solely of 3T3-L1 cells in a concentration range between 1 nM and 1 μM by up to 8.84 ± 4.97 or 7.49 ± 5.08%, respectively. Blockage of TRPV1 using the specific inhibitor *trans-tert*-butyl-cyclohexanol demonstrated that the anti-adipogenic activity of *trans-*pellitorine depends on TRPV1. In addition, blockage of the TRPA1 channel using the antagonist AP-18 showed a TRPA1-dependent signaling in the early to intermediate stages of adipogenesis. On a mechanistic level, treatment with *trans-*pellitorine during adipogenesis led to reduced PPARγ expression on gene and protein level via activation of TRPV1 and TRPA1, and increased expression of the microRNA *mmu-let-7b*, which has been associated with reduced PPARγ levels. In addition, cells treated with *trans*-pellitorine showed decreased expression of the gene encoding for fatty acid synthase, increased expression of *microRNA-103* and a decreased short-term fatty acid uptake on the functional level. In summary, these data point to an involvement of the TRPV1 and TRPA1 cation channels in the anti-adipogenic activity of *trans-*pellitorine via *microRNA-let7b* and PPARγ. Since *trans*-pellitorine does not directly activate TRPV1 or TRPA1, an indirect modulation of the channel activity is assumed and warrants further investigation.

## Introduction

Adipose tissue has been originally considered to be an inert tissue that stores fat, although, over the past decades, it has been shown to be a metabolically highly active endocrine organ as well (Coelho et al., 2013). The major cell type of adipose tissue are adipocytes, which are the result from differentiation of preadipocytes to mature adipocytes in a process called adipogenesis (Hausman et al., 2001). Preadipocytes are capable of differentiation to adipocytes throughout life (Hausman et al., 2001), which can result in hyperplastic expansion of adipose tissue (Coelho et al., 2013). Pathological overgrowth of visceral adipose tissue is associated with factors determining the metabolic syndrome such as overweight and insulin resistance (Kivipelto et al., 2005). Since about 10% of the adipocytes are renewed every year in adults, targeting the process of adipogenesis to support maintenance of a healthy body weight composition has been in focus of research in the past years (Bray and Tartaglia, 2000).

Promising results have been demonstrated for several phytochemicals, such as the dietary bioactives genistein (Zhang et al., 2009), resveratrol (Rayalam et al., 2008), and quercetin (Ahn et al., 2008). Also the alkaloids capsaicin and its structural analog nonivamide present in *Capsicum* species show anti-adipogenic activity *in vitro* and *in vivo*, which has been associated with activation of the transient receptor potential vanilloid type-1 (TRPV1) (Hsu and Yen, 2007; Zhang et al., 2007; Rohm et al., 2015; Hochkogler et al., 2017). However, activation of TRPV1 does not only inhibit adipogenesis, but also induces a pungent sensation in the oral cavity, which is limiting the dietary intake (Ursu et al., 2010). Another phytochemical that is known for its chemesthetic properties and used as an aroma compound is *trans-*pellitorine. As an alkamide, *trans-*pellitorine [(2*E*,4*E*)-decadienoic acid *N*-isobutyl amide] shares some structural motifs with the capsaicinoids capsaicin and nonivamide, but is lacking the vanillyl-goup. *trans-*pellitorine is sensorially not described as pungent but induces a tingling effect in the oral cavity (Obst et al., 2017). This is congruent with a recent study from our own group which demonstrates that *trans-*pellitorine is not directly activating TRPV1 (Walker et al., 2017). Nevertheless, an involvement of TRPV1 in the signaling cascade of *trans-*pellitorine cannot be excluded, since it has been demonstrated that the anti-inflammatory properties of *trans-*pellitorine in U937 macrophages can be blocked by addition of a TRPV1 antagonist (Walker et al., 2017). However, anti-inflammatory responses of a *trans-*pellitorine-treatment could also be blocked by a TRPA1 antagonist (Walker et al., 2017), suggesting TRPA1-involvement in *trans-*pellitorine signaling as well. Furthermore, the TRPA1 agonist cinnamaldehyde has been associated with reduced visceral body fat in mice (Tamura et al., 2012), associating also TRPA1 signaling with anti-obesity effects. Thus, we hypothesize that the tingling alkamide *trans-*pellitorine may influence adipogenesis, possibly via TRPV1 and TRPA1 signaling.

To investigate this hypothesis, we assessed lipid accumulation of the well-defined adipocyte-model 3T3-L1 cells after application of *trans-*pellitorine during differentiation and maturation. Adipogenesis of 3T3-L1 preadipocytes to fully mature adipocytes is well-investigated: contact inhibition after reaching confluence leads to growth arrest followed by mitotic clonal expansion via the application of a hormone cocktail containing insulin, cAMP analogs and glucocorticoids. After these very early stages of adipogenesis, replication of preadipocytes starts before the cells are terminally differentiated to adipocytes (Gregoire et al., 1998). This process is regulated by several transcription factors, amongst others peroxisome-proliferator-activated receptor gamma (PPARγ), CCAAT-enhancer-binding protein (C/EBP) α, β, and δ (Rosen and Spiegelman, 2000; Farmer, 2006). In addition, a regulation of this process by several microRNAs (miRNA) has been demonstrated as well (McGregor and Choi, 2011). miRNAs are non-coding RNAs with about 20 bp in length that regulate expression of target mRNA, thus controlling physiological processes including the differentiation of preadipocytes (Eulalio et al., 2008). For example, several miRNAs like miR-103, miR-143, and members of the let-7 group have been shown to reduce adipogenesis by targeting the transcription factor PPARγ (Esau et al., 2004; Sun et al., 2009; Xie et al., 2009). Recently, we demonstrated that miRNA expression in differentiating 3T3-L1 cells is a target of the TRPV1 agonist nonivamide and showed an involvement of miRNA let-7d in the regulation of 3T3-L1 differentiation by nonivamide (Rohm et al., 2015).

In order to clarify the mechanism by which *trans-*pellitorine might impact differentiation and maturation of 3T3-L1, targets of pellitorine in the signaling cascade of adipogenesis were identified by investigation of miRNA expression and target mRNA analysis, as well as PPARγ and fatty acid synthase (FAS) protein levels. In addition, an involvement of the cation channels TRPV1 and TRPA1 in *trans-*pellitorine-signaling was investigated by application of the TRPV1-antagonist *trans*-*tert*-butylcyclohexanol (BCH) and the TRPA1 antagonist AP-18.

## Materials and Methods

### Materials

*trans*-Pellitorine and *trans-tert*-butylcyclohexanol (BCH) were kindly provided by Symrise AG; *trans*-pellitorine had a purity of >95% (GC). 3T3-L1 fibroblasts were purchased from ATCC (CL-173^TM^). All other reagents were purchased from Sigma Aldrich (Austria), unless stated otherwise.

The test compounds *trans-*pellitorine, BCH and AP-18 were dissolved in ethanol (1000 × concentrated) freshly before each experiment. The final concentration of ethanol during the incubations never exceeded 0.2%.

### Cell Culture

3T3-L1 preadipocytes were maintained in high-glucose DMEM (4.5 g/L glucose) supplemented with 10% heat-inactivated FBS (Thermo Fisher Scientific, Austria), 4 mM L- glutamine and 1% penicillin (10,000 U)/streptomycin (10 mg/mL) at 37°C and 5% CO_2_ in a humidified incubator. Cells were split at ∼70–80% confluence and used between passages 6 and 22.

Differentiation into adipocytes was carried out as described before (Rohm et al., 2015). Only monolayers with a differentiation grade of at least ∼85% were used for the studies, which was assured using microscopy.

### Cell Viability

Negative effects of the applied concentrations of *trans*-pellitorine with or without addition of BCH or AP-18 were excluded by analyzing cell proliferation as a measure for cell viability using MTT [3-(4,5-dimethylthiazol-2-yl)-2,5-diphenyltetrazolium bromide] (Carl Roth, Germany) dye in 96-well format as described previously (Rohm et al., 2015).

### Oil Red O Staining

The accumulation of lipids during adipogenesis and maturation of 3T3-L1 adipocytes was analyzed by means of oil Red O staining as described before (Rohm et al., 2015). Briefly, cells were kept in maturation medium for 10 days, and medium was renewed every 48 h before cells were fixated with formalin solution with a final concentration of 4% (v/v) formalin in PBS. Substance addition (*trans*-pellitorine with or without addition of BCH/AP-18) was started on day 0 (differentiation) or day 2 (maturation) after induction of differentiation by addition of the hormone cocktail. An impact of *trans*-pellitorine with or without addition of BCH and AP-18 on lipid accumulation was calculated in % relative to vehicle (0.1–0.2% ethanol) treated cells.

### Fatty Acid Uptake

Uptake of BODIPY-C_12_ was analyzed as a measure of short-term free fatty acid uptake using the QBT^TM^ fatty acid uptake kit (Molecular Devices, Germany) as described in detail before (Holik et al., 2016) in fully differentiated 3T3-L1 adipocytes on day 9 after induction of differentiation. Cells were pre-treated with 10 nM–10 μM *trans-*pellitorine for 30 min at 37°C and 5% CO_2_ before the fatty acid uptake reaction was started by addition of loading dye. The uptake of BODIPY-C_12_ was recorded every 20 s for a total of 60 min with an emission wavelength of 515 nm and an excitation wavelength of 485 nm. The area under the curve (AUC) of the corresponding time/emission plots was used for data calculation.

### ddPCR

Absolute quantification of miRNA expression levels was carried out using the Bio-Rad QX200 Droplet Digital PCR System as described before (Rohm et al., 2015). In brief, miRNA from undifferentiated control cells (day 0) or mature adipocytes treated with 1 μM *trans*-pellitorine or 0.1% ethanol (day 9) was isolated using the RNeasy Lipid Tissue Mini Kit (Qiagen, Austria) using wash buffer RWT instead of wash buffer RW1 to preserve smaller RNA pieces. Reverse transcription to cDNA was carried out using specific primers for the target miRNA (Life technologies/Thermo Fisher) in combination with the TaqMan MicroRNA Reverse Transcription Kit (Life technologies/Thermo Fisher). cDNA was mixed with droplet PCR supermix (Bio-Rad, Germany) and each sample was partitioned into 20,000 single droplets using the droplet generator before PCR was carried out using a C1000 thermocycler (Bio-Rad, Germany). Droplets were analyzed by means of a QX200 droplet reader (Bio-Rad, Germany) and absolute concentrations (copies/μL) were computed with the QuantaSoft software. The non-coding U6 snRNA was used for normalization (Chen et al., 2016) and data are calculated as fold change to undifferentiated or vehicle-control treated cells as indicated.

### Quantitative Real Time-Polymerase Chain Reaction

Gene expression of *PPARG, FAS, LRP2*, and *IGF1R* in pellitorine-treated 3T3-L1 adipocytes was analyzed by quantitative Real-Time PCR. RNA was isolated on day 0 (before initiation of differentiation) and day 9 after initiation of differentiation with or without treatment with 1 or 10 μM *trans-*pellitorine using the RNeasy Lipid tissue Mini Kit (Qiagen) according to manufacturer’s instructions. Quality and concentration of the isolated RNA was analyzed by absorbance using a NanoQuant Plate (Tecan, Austria) and an Infinite M200 Plate reader (Tecan) before the RNA was reversely transcribed to cDNA using the high capacity RNA to cDNA Kit (ABI, Thermo Fisher). Fluorescence intensities during PCR were monitored in triplicates using Fast SYBR Green Master Mix (ABI, Thermo Fisher) on a StepOnePlus device (ABI, Thermo Fisher). Nucleotide sequences, product lengths and references for the primers used in the reactions can be found in **Table 1**. All primers were synthesized by Sigma Aldrich, Austria. Hypothetical mRNA starting concentrations were calculated using LinReg v12.8, normalized to hypoxanthine guanine phosphoribosyl transferase (*HPRT1*) as endogenous control (Han et al., 2002; Diaz-Villasenor et al., 2013; Rohm et al., 2015; Holik et al., 2016) and are given as fold change to vehicle control treated cells (=1).

**Table 1:** Oligonucleotide sequences used during PCR.

Target	Forward primer	Reverse primer	Product length [bp]	Reference
*HPRT*	GAGAGCGTTGGGCTTACCTC	ATCGCTAATCACGACGCTGG	136	Rohm et al., 2015
*PPARG*	GTGCCAGTTTCGATCCGTAGA	GGCCAGCATCGTGTAGATGA	142	Rohm et al., 2015
*FAS*	CACAGATGATGACAGGAGATGG	TCGGAGTGAGGCTGGGTTGAT	205	Hewitt et al., 2004
*LRP2*	TGTACCAGAAAATGTGGAAAACC	GTGTCTTCTGTGGCAGTGTAGC	288	Lyons et al., 2003
*IGF1R*	GGAGTGTCCCTCAGGCTTCA	CATCGCCGCAGACTTTGG	89	Nef et al., 2003

### Determination of PPARγ and FAS Protein Levels

PPARγ and FAS protein levels were quantified using specific ELISA Kits (mouse PPARγ, mouse FASN, Cloude-Clone, Corp., United States, with a sensitivity of 0.66 ng/mL for PPARγ and 0.057 ng/mL for FAS). To obtain protein lysates for sampling, undifferentiated (day 0) or mature 3T3-L1 adipocytes (day 9, treated or untreated with 1 or 10 μM *trans-*pellitorine) were washed twice with ice-cold PBS and harvested in lysis buffer (50 mM Tris, 25 mM NaCl, 1 mM EDTA, 1 mM NaF, 1% of the non-denaturing detergent Igepal, pH 7.4) supplemented with 1 mM PMSF, 1 mM sodium ortho-vanadate and protease inhibitor cocktail.

The lysates were passed several times through a 20-gauge needle (Sterican, B.Braun Melsungen AG, Germany) and homogenized under gentle agitation for 30–45 min at 4°C before a high-speed centrifugation step (16,900 × *g* for 15 min at 4°C) was applied. The supernatant was assayed on the respective ELISA as recommended by manufacturer’s protocol. PPARγ and FAS content were normalized to the protein content of each sample, which was determined via Bradford. Data are calculated as percentage of the corresponding vehicle control.

### Statistical Analysis

Data are presented as (difference in) mean fold changes, or (differences in) mean fold change in percent compared to the corresponding controls ± SEM. All data are calculated from at least three independent experiments with multiple technical replicates each as indicated in the figure or table legends, where n refers to the number of biological replicates. Since the test compounds *trans-*pellitorine and BCH had to be dissolved in ethanol as 1000 × stock solutions, an effect of 0.1–0.2% ethanol in the different assays was excluded and the data are calculated in comparison to the corresponding vehicle control. Outliers were determined using Nalimov outlier test and excluded from further calculations. Data sets were tested for normal distribution and equal variances using Shapiro–Wilk test and Levene–Median test, respectively. Significant differences between two groups were determined by applying Student’s *t*-test or Mann–Whitney *U* test in case of not-normally distributed data sets or unequal variances. Significant differences between multiple groups were assessed using one-way ANOVA with Holm–Sidak *post hoc* test, or one-way ANOVA on Ranks followed by Dunn’s *post hoc* test for not-normally distributed data sets or unequal variances. A *p*-value of <0.05 was considered as statistically significant. Statistical analysis was carried out using SigmaPlot 11.0 or SigmaPlot 13.0. Statistical significant differences between the groups are marked in the figures by distinct letters and ^∗^*p* < 0.05, ^∗∗^*p* < 0.01, ^∗∗∗^*p* < 0.001 vs. control.

## Results

### Cell Viability

Potential negative effects on cell viability of the applied concentrations of *trans*-pellitorine with or without addition of BCH or AP-18 for 12 days were determined by analyzing cell proliferation as a measure for cell viability using the MTT assay. The number of metabolically active cells after treatment with 0.001–10 μM *trans-*pellitorine, 25 μM BCH, 1 μM AP-18 or a combination of 1 μM *trans-*pellitorine and 25 μM BCH or 1 μM AP-18, was not reduced in comparison to non- or vehicle control-treated cells (data not shown, *p* > 0.05).

### Treatment with *trans-*Pellitorine during Adipogenesis of 3T3-L1 Cells Reduced Lipid Accumulation

**FIGURE 1:**
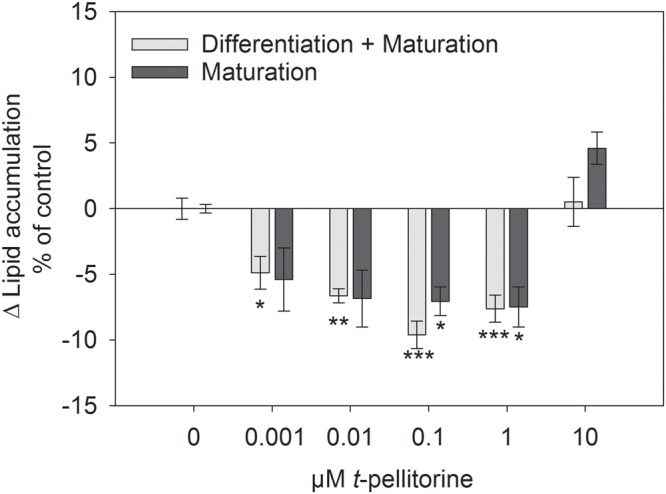
Difference in lipid accumulation in % of control (0.1% EtOH) ± SEM after addition of 0.001–10 μM *trans-*pellitorine during differentiation and maturation for 12 days (light gray bars) or during maturation for 10 days (dark gray bars) after initiation of differentiation of 3T3-L1 cells. Lipids were stained using oil red O dye and the results are shown as the mean difference of control treated cells from three to four independent experiments with multiple technical replicates. Significant differences to the corresponding control treated cells were analyzed by one-way ANOVA with Holm–Sidak *post hoc* test and are marked with ^∗^*p* < 0.05, ^∗∗^*p* < 0.01, and ^∗∗∗^*p* < 0.001 vs. the corresponding controls.

The accumulation of lipids analyzed by Oil Red O staining is a frequently used marker for the degree of differentiation of 3T3-L1 cells (Arumugam et al., 2008). In the present study, we assessed lipid accumulation of 3T3-L1 cells by this staining method when treated with 1 nM to 10 μM *trans-*pellitorine (1) during the whole differentiation process, corresponding to 12 days of treatment, and (2) during the maturation phase solely, corresponding to 10 days of treatment. An effect of the vehicle ethanol (0.1–0.2%, EtOH control) was excluded in a previous study (Rohm et al., 2015). The data depicted in **Figure 1** (light gray bars) show that the addition of *trans-*pellitorine in a concentration between 1 nM and 1 μM during differentiation led to a reduced lipid accumulation by up to 9.61 ± 1.05% at a concentration of 0.1 μM (*p* < 0.001 vs. EtOH control). However, also the addition of *trans-*pellitorine during the maturation phase solely reduced lipid accumulation to a similar extent by up to 7.49 ± 1.53% at a concentration of 1 μM (*p* < 0.001 vs. EtOH control). A concentration of 10 μM had no effect on lipid accumulation, independent of the administration during differentiation or maturation (*p* > 0.05 vs. EtOH control).

### Administration of *trans-*Pellitorine Affects Short-Term Fatty Acid Uptake

**FIGURE 2:**
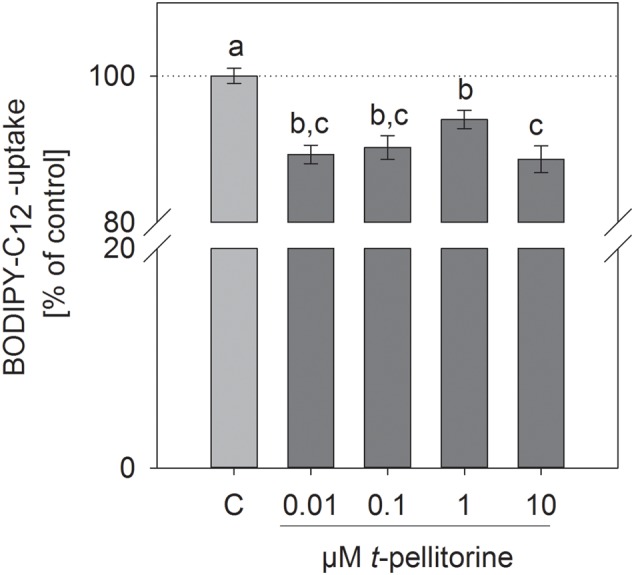
Effect of 0.01–10 μM *trans-*pellitorine on Bodipy-C_12_ uptake as a measure of fatty acid uptake in fully differentiated 3T3-L1 cells. Cells were pre-treated with different concentrations of *trans*-pellitorine for 30 min. Treatment with 100 nM insulin for 30 min was used as a positive control and increased fatty acid uptake to 139 ± 11.7% (*p* < 0.001, not shown in the figure). Statistics: mean ± SEM, *n* = 6 with three technical replicates each. Significant differences (*p* < 0.05) were assessed by one-way ANOVA with Holm–Sidak *post hoc* test and are marked with distinct lower-case letters.

We investigated whether the reduction of lipid accumulation during the maturation of 3T3-L1 cells may be due to a reduced fatty acid uptake. Pre-treatment for 30 min of fully differentiated 3T3-L1 cells with 0.001–10 μM *trans-*pellitorine for 30 min reduced fatty acid uptake, which is depicted in **Figure 2**. The most pronounced effect was detected after incubation with 10 μM, which decreased fatty acid uptake to 88.6 ± 1.84% (*p* < 0.001). Treatment with 100 nM insulin was used as a positive control and enhanced fatty acid uptake to 139 ± 11.7% (*p* < 0.001, data not shown in figure).

### Treatment with *trans-*Pellitorine Regulates miRNA Expression

Several miRNAs have been demonstrated to be involved in the control of adipogenesis and obesity (see review Romao et al., 2011). Recently, we identified the miRNAs *let-7a, let-7b, let-7d, miR-103*, and *miR-143* as potential targets of TRPV1-dependent inhibition of adipogenesis by the pungent compound nonivamide using a customized miRNA-array (Rohm et al., 2015). Thus, in the present study, we investigated the effect of an addition of 1 μM *trans-*pellitorine to developing adipocytes on the expression of the named miRNAs by means of the highly sensitive digital-droplet PCR. A concentration of 1 μM *trans-*pellitorine was chosen since the greatest effect size on maturation was detected using this concentration. In addition, *trans-*pellitorine-treatment led to reduced lipid accumulation at the lower test concentrations of 1 and 10 nM only when added during the early to intermediate stages of adipogenesis. Thus, to obtain the highest effect size, the following experiments were carried out after treatment during differentiation and maturation phase. **Table 2** shows that, in comparison to undifferentiated control cells, expression levels of *mmu-miR-let-7a, mmu-miR-let-7b, mmu-let-7d, mmu-miR-103*, and *mmu-miR-143* were largely increased in fully differentiated 3T3-L1 adipocytes and that addition of 0.1% ethanol during adipogenesis and maturation did not impact the expression of *mmu-miR-let-7a, mmu-miR-let-7b, mmu-let-7d, mmu-miR-103*, and *mmu-miR-143*. However, addition of 1 μM *trans-*pellitorine, which significantly decreased lipid accumulation when added to developing 3T3-L1 cells, increased expression of *mmu-miR-let-7b* to fold changes of 1.53 ± 0.31, and *miR-103* to 1.50 ± 0.25 vs. vehicle control treated cells (data not shown in figure or table). Fold changes (*trans*-pellitorine/vehicle control) of ≥1.5 or ≤0.5 were used as cut-off criteria for regulation according to Li et al. (2011). Expression levels of *mmu*-*miR-let-7a* (1.19 ± 0.06), *mmu-miR-let-7d* (1.40 ± 0.16), and *mmu-miR-143* (1.46 ± 0.12) after treatment with 1 μM *trans*-pellitorine did not meet these cut-off criteria (data not shown in figure or table) and were not further investigated.

**Table 2:** Results of the ddPCR analysis of selected miRNAs in mature 3T3-L1 adipocytes (untreated or vehicle control/0.1% EtOH- treated cells) in comparison to undifferentiated control cells.

	Control	EtOH control
*miR-let-7a*	6.20 ± 0.45	6.58 ± 1.70
*miR-let-7b*	26.5 ± 6.70	27.2 ± 8.04
*miR-let-7d*	9.80 ± 1.87	11.1 ± 3.06
*miR-103*	19.8 ± 3.87	24.1 ± 5.10
*miR-143*	29.2 ± 1.13	27.6 ± 6.17

*A fold change of 0.5 or 1.5 respectively, was used as cut-off criteria for regulation. Fold change ± SEM of selected microRNA expression on day 9 after induction of differentiation vs. undifferentiated control cells (=1) analyzed by means of digital droplet PCR. *n* = 3.*

### Treatment with *trans*-Pellitorine Decreases Expression and Protein Levels of PPARγ

The online-tool TargetScanMouse by the Whitehead Institute for Biomedical Research^1^ was used to select mRNA targets of the regulated miRNAs *miR-let-7b* and *miR-103* which may be involved in adipogenesis, obesity and lipid metabolism. As mRNA targets of *mmu-miR-let-7b*, genes encoding for the transcription factor peroxisome proliferator-activated receptor gamma (*PPARG)* and the growth-promoting insulin-like growth factor 1 receptor (*IGF1R*) were chosen for further investigation using quantitative Real-Time PCR. As targets of *mmu-miR-103*, genes encoding for *FAS* and low density lipoprotein-related protein 2 (*LPR2*) were chosen.

**FIGURE 3:**
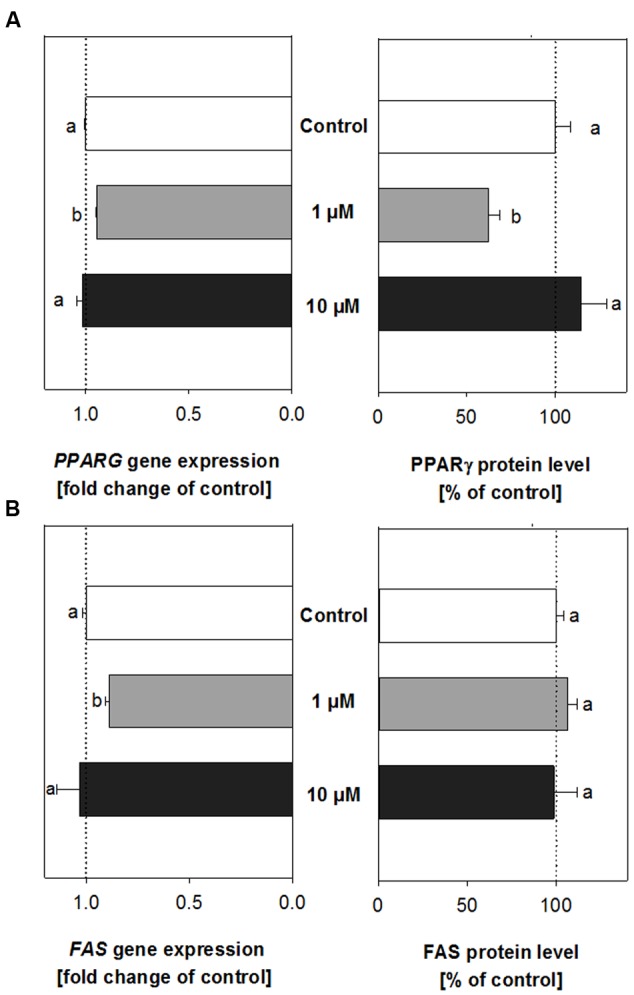
PPARγ **(A)** and fatty acid synthase (FAS) **(B)** gene expression and protein levels in cells treated with either 1 μM (gray bars) or 10 μM (black bars) *trans-*pellitorine in comparison to control (0.1% EtOH) treated cells (white bars). Statistics: differences in *PPARG* and *FAS* gene expression were assessed using one-way ANOVA on Ranks with Dunn’s *post hoc* test, *n* = 3–4 with three technical replicates each. Significant differences (*p* < 0.05) in PPARγ and FAS protein levels were analyzed with one-way ANOVA and Holm–Sidak *post hoc* test (*n* = 3–5, with two technical replicates) or one-way ANOVA on Ranks with Dunn’s *post hoc* test, respectively; *n* = 4 with two technical replicates. Significant differences between the groups are marked with distinct lower-case letters.

Treatment of developing adipocytes with 1 μM *trans-*pellitorine did not change mRNA expression of *IGF1R* and *LRP2* (data not shown). However, we detected decreased mRNA expression levels for *PPARG* (**Figure 3A**, left side) and *FAS* (**Figure 3B**, left side). *PPARG* expression was slightly reduced to 0.95 ± 0.004 (*p* < 0.001), and also *FAS* expression decreased to 0.89 ± 0.02 (*p* < 0.01). No altered expression of *PPARG* and *FAS* was detected after treatment with 10 μM *trans-*pellitorine (**Figures 3A** + B, left side), the same concentration that did not result in decreased lipid accumulation as well. The results of the mRNA expression of genes encoding for PPARγ and FAS after treatment with either 1 or 10 μM *trans-*pellitorine were verified on protein level.

In contrast to the decreased mRNA expression after treatment with 1 μM *trans-*pellitorine, protein levels of FAS were neither altered after treatment with 1 μM nor with 10 μM *trans-*pellitorine (**Figure 3B**, right side). However, protein levels of PPARγ were, in accordance with the mRNA expression levels, reduced after treatment with 1 μM by 37.6 ± 6.44% (*p* < 0.05), but not after treatment with 10 μM *trans-*pellitorine.

### Involvement of TRPA1 and TRPV1 in the Anti-adipogenic Effects of *trans-*Pellitorine

**FIGURE 4:**
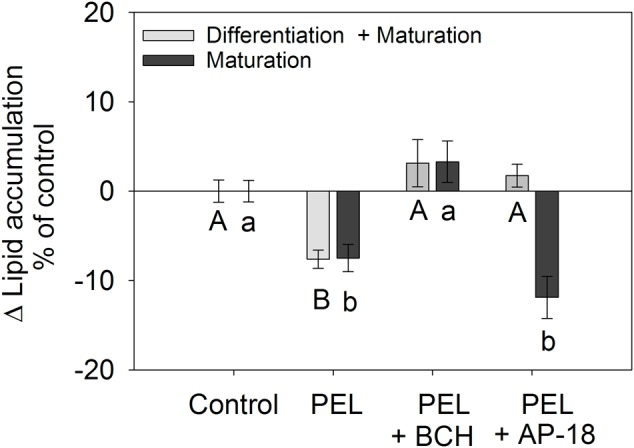
Difference in lipid accumulation in % of control (0.1% EtOH) ± SEM after addition of 1 μM *trans-*pellitorine (PEL) with or without the addition of 25 μM of the TRPV1 antagonist BCH or 1 μM of the TRPA1 antagonist AP-18 during differentiation and maturation for 12 days (light gray bars) or during maturation for 10 days (dark gray bars) after initiation of differentiation of 3T3-L1 cells. Lipids were stained using oil red O dye and the results are shown as the mean difference of control treated cells from 3 to 6 independent experiments with multiple technical replicates. Significant differences (*p* < 0.05) to the corresponding control treated cells were analyzed by one-way ANOVA on Ranks with Dunn’s *post hoc* test and are marked with distinct capital letters for the treatments during differentiation + maturation phase and lower-case letters for the treatments during maturation phase.

The cation channel TRPV1 has been shown to be involved in the regulation of adipogenesis by capsaicin (Zhang et al., 2007) and nonivamide (Rohm et al., 2015). Additionally, also the TRPA1 agonist cinnamaldehyde has been associated with reduced visceral body fat in mice (Tamura et al., 2012) and decreased lipid accumulation in 3T3-L1 cells (Khare et al., 2016). In order to examine whether the reduced lipid accumulation after treatment with the trigeminally active compound *trans*-pellitorine is associated with activation of the TRPV1 or TRPA1 channel as well, 3T3-L1 cells were co-incubated with 1 μM *trans*-pellitorine and 25 μM of the TRPV1 inhibitor *trans*-*tert*-butylcyclohexanol (BCH) or 1 μM of the TRPA1 inhibitor AP-18 for 10 or 12 days, respectively. A concentration of 1 μM *trans-*pellitorine was chosen for the co-incubation studies since it demonstrated the largest effect size when cells were treated during maturation in the previous experiments. The inhibitor BCH has been shown to be effective in blocking the effect of capsaicin and nonivamide on lipid accumulation assessed by oil red O staining in a previous study (Rohm et al., 2015). AP-18 was applied at a concentration of 1 μM since this concentration has been shown to block TRPA1-mediated responses in HEK-293 cells (Birrell et al., 2009). Higher concentrations of 10 μM AP-18 in combination with 1 μM *trans*-pellitorine significantly reduced metabolic activity to 90.3 ± 2.58% (*p* < 0.01, data not shown in figure) according to the MTT assay in 3T3-L1 cells and were therefore not applied. Addition of 25 μM of the inhibitor BCH diminished the inhibiting effect of 1 μM *trans-*pellitorine on lipid accumulation, leading to no difference in lipid accumulation relative to control treated cells (**Figure 4**). No difference was detected between the concomitant administration of *trans-*pellitorine and BCH during the whole adipogenesis (12 days) or during the maturation phase (10 days). Addition of the TRPA1 inhibitor AP-18 (1 μM) to *trans*-pellitorine (1 μM)-containing medium diminished the inhibiting effect of *trans*-pellitorine on lipid accumulation (+1.00 ± 1.27%, *p* < 0.01 vs. *trans-*pellitorine, *p* > 0.05 vs. control) when added during the whole differentiation for 12 days to control level. However, addition of 1 μM AP-18 did not affect the effect of *trans-*pellitorine when added during the maturation phase for 10 days (-11.9 ± 2.36%, *p* < 0.001 vs. control, *p* > 0.05 vs. *trans-*pellitorine, **Figure 4**).

**FIGURE 5:**
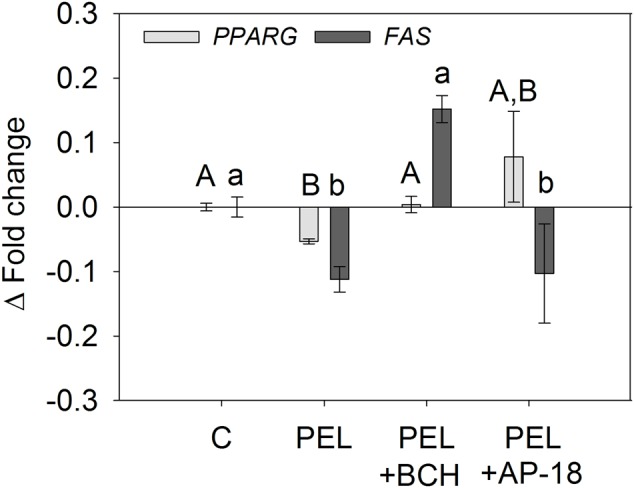
*PPARG* and *FAS* gene expression after treatment with 1 μM *trans-*pellitorine (PEL) without or with addition of 25 μM of the TRPV1 antagonist BCH or 1 μM of the TRPA1 antagonist AP-18 during differentiation and maturation of 3T3-L1 preadipocytes. Statistics: *n* = 4 with three technical replicates each. Significant differences (*p* < 0.05) in gene expression between the treatments were analyzed using one-way ANOVA on Ranks with Dunn’s *post hoc* test and are marked with distinct letters capital letters for differences in the gene expression levels of *PPARG* and lower-case letters for differences in the gene expression of *FAS*.

In a next step, we assessed whether the decreased mRNA expression of *PPARG* and *FAS* is associated with TRPV1 and TRPA1 activation by means of an addition of the TRPV1 inhibitor BCH and the TRPA1 inhibitor AP-18 as described above. In accordance with the results from the lipid accumulation experiments, addition of 25 μM BCH diminished the effect of 1 μM *trans-*pellitorine, with mRNA expression similar to control level (1.00 ± 0.01 for *PPARG* and 1.15 ± 0.08 for *FAS*, **Figure 5**). Furthermore, addition of 1 μM AP-18 diminished the effect of 1 μM *trans-*pellitorine on *PPARG* expression, bringing it back to control level (1.08 ± 0.07), but did not impact *FAS* gene expression in comparison to a treatment with *trans-*pellitorine solely (0.90 ± 0.02), as depicted in **Figure 5**.

## Discussion

The phytochemicals capsaicin and nonivamide, present in *Capsicum* species, have been demonstrated to reduce adipogenesis in 3T3-L1 cells when applied in a nanomolar to micromolar range (Rohm et al., 2015). The usage of inhibitors and knock-out models demonstrated an involvement of the TRPV1 cation channel (Zhang et al., 2007; Rohm et al., 2015). However, the activation of the TRPV1 channel by capsaicinoids is also responsible for the sharp burning chemesthetic (trigeminal) sensation in the oral cavity, which is often referred to as pungency and limits the dietary intake of those compounds (Ursu et al., 2010). Another phytochemical with chemesthetic properties is *trans-*pellitorine. *trans-*pellitorine is lacking the vanillyl-group, but shares the amide group coupled to a carbon chain with the TRPV1 agonists capsaicin and nonivamide. The alkamide *trans-*pellitorine present in *Piper* and *Macropiper* species is known for its tingling effects (Obst et al., 2017), which is why the aroma compound has been discussed as a TRPV1 and also TRPA1 agonist as well (Sharma et al., 2011; Boonen et al., 2012). However, a recent study from our own group showed that *trans-*pellitorine does not directly activate TRPV1 and TRPA1, although both TRP channels seemed to be involved in the signaling cascade of the anti-inflammatory activity of *trans-*pellitorine in U937 macrophages (Walker et al., 2017). Thus, in the present study, we hypothesized that *trans-*pellitorine may influence adipogenesis, possibly via TRPV1, as well.

As a model, well-defined 3T3-L1 preadipocytes were chosen and treated with *trans-*pellitorine in concentrations ranging between 0.001 and 10 μM to fully mature adipocytes during early differentiation and/or maturation. Lipid accumulation was assessed using oil red O staining, which is a frequently used marker for adipogenesis in 3T3-L1 cells (Ahn et al., 2008; Zhang et al., 2009; Rohm et al., 2015; Lim et al., 2016). Lipid accumulation was reduced after application of 0.001 to 1 μM *trans-*pellitorine, but not after 10 μM. This indicates a potential biphasic effect of *trans-*pellitorine on lipid accumulation in developing 3T3-L1 cells which will be discussed in more detail below. Furthermore, two modes of application of *trans-*pellitorine were investigated in the present study: an application during early differentiation and maturation for 12 days and an application during maturation, the later stages of differentiation for 10 days. Application of *trans-*pellitorine during differentiation and maturation (12 days) lowered the degree of lipid accumulation as a marker for differentiation to a similar extent as an addition during the maturation (10 days) phase alone at the higher test concentrations starting from 100 nM. This is pointing to an effect of *trans-*pellitorine on the intermediate to later stages of the differentiation to fully mature adipocytes rather than to an effect on the very early stages like clonal expansion (Ntambi and Young-Cheul, 2000).

During the later stages of adipogenesis, 3T3-L1 cells accumulate triglycerides and other lipids (Coelho et al., 2013). We investigated whether fatty acid uptake in 3T3-L1 cells is differently influenced by concentrations of *trans-*pellitorine ranging between 0.01 and 10 μM. *trans-*pellitorine reduced short-term fatty acid uptake after 30 min pretreatment with 0.01–1 μM. Remarkably, in contrast the lipid accumulation, also a concentration of 10 μM significantly reduced fatty acid uptake. Thus, the decreased fatty acid uptake may contribute to the decreased lipid accumulation in lower concentrations, but does not explain the difference in the response between lower and higher concentration of *trans-*pellitorine. Therefore, as a next step, we aimed to clarify if the addition of *trans-*pellitorine has an impact on the molecular level of adipogenesis, the gene expression. To investigate which genes may be involved in the effects of *trans-*pellitorine on lipid accumulation, we screened the expression of certain miRNAs that have been selected according to our previous studies at which miRNA-arrays detecting expression levels of all known mature mouse miRNAs were applied (Rohm et al., 2015; Holik et al., 2016). In detail, we investigated expression of *miR-let-7a, let-7b, let-7d, miR-103*, and *miR-143* after treatment with 1 μM *trans-*pellitorine. The results present evidence that expression levels of *mmu-miR-let-7a, mmu-miR-let-7b, mmu-let-7d, mmu-miR-103*, and *mmu-miR-143* are increased during adipogenesis and maturation of 3T3-L1 cells, supporting an effect of the selected miRNAs on adipogenesis of 3T3-L1 cells. An effect of the selected miRNAs on adipogenesis has been demonstrated by other groups as well (Esau et al., 2004; Wilfred et al., 2007; Sun et al., 2009).

Furthermore, the present study shows that expression of the miRNAs *mmu-miR-103* and *mmu-miR-let-7b* is upregulated after treatment with 1 μM *trans-*pellitorine. miRNA usually have more than one target gene (Lewis et al., 2005). We selected target genes that affect intermediate to later stages of adipogenesis according to the database TargetScanMouse provided by the Whitehead Institute for Biomedical Research. As target genes for mmu-miR-103, *FAS* and *LPR2* were chosen, and for *mmu-miR-let-7b, PPARG* and *IGF1R* were selected. While expression levels of *LPR2* and *IGF1R* were not altered by treatment with 1 μM *trans-*pellitorine, expression of *PPARG* and *FAS* was slightly but significantly downregulated. PPARγ is a highly important transcription factor that regulates the intermediate stages of adipogenesis by starting a transcription cascade of adipogenic genes. *PPARG* gene expression is highly upregulated during adipogenesis after the clonal expansion, starting about 24–48 h after induction of differentiation by addition of IBMX (Burton et al., 2004). In contrast, *FAS* is a marker of the later stages of adipogenesis and its expression is robustly increased about 5 days after induction of differentiation (Habinowski and Witters, 2001). To clarify whether the different effects of 1 and 10 μM *trans-*pellitorine can be detected on the molecular level as well, expression of *PPARG* and *FAS* were investigated after treatment with 10 μM as well. As expected, expression of both genes was not altered. As a next step, we addressed changes of PPARγ and FAS on the protein level after treatment with *trans-*pellitorine. While FAS protein levels were not different compared to control level after treatment with either 1 or 10 μM *trans-*pellitorine, PPARγ protein levels were decreased after treatment with 1 μM, but not 10 μM *trans-*pellitorine, which is in accordance with the gene expression data. Thus, PPARγ may be a target of *trans-*pellitorine’s anti-adipogenic activity, which is only present at concentrations up to 1 μM.

Structurally related alkamides with 12–16 C-atoms in the alkyl chain have been shown to increase rather than decrease lipid accumulation in 3T3-L1 cells accompanied by increased PPARγ protein levels when applied in concentrations of 30–50 μM (Christensen et al., 2009; Shin et al., 2014). In addition, the study results from Christensen et al. (2009) indicated that PPARγ stimulation by alkamides had the highest affinity for long-chained alkamides with 16–20 carbon atoms. However, medium-chain alkamides like *trans*-pellitorine (10 carbon atoms) were not yet included in these studies. In addition, the PPARγ-stimulating effects of the tested alkamides were demonstrated only at concentrations that are at least three-times higher than used in the present study. *trans-*pellitorine showed a dose-related effect on lipid accumulation, although, to clarify a dose-dependency of *trans-*pellitorine and related alkamides, a higher range of test concentrations would be needed.

The effect of the other trigeminally active compounds nonivamide and capsaicin on lipid accumulation in 3T3-L1 cells have been associated with an activation of the TRPV1 cation channel with an involvement of PPARγ as well (Rohm et al., 2015). In detail, the following signaling pathway for capsaicin is proposed: activation of TRPV1 is thought to increase intracellular calcium levels that target adjacent calcineurin, which is thought to inhibit the pro-adipogenic factors PPARγ and C/EBPα (Cioffi, 2007). *trans-*pellitorine has been demonstrated to increase intracellular calcium-mobilization in neural cells and, in addition, it has been shown that TRPV1 and also TRPA1 may play a role in the signaling pathway of *trans-*pellitorine in other cell models (Rohm et al., 2014; Walker et al., 2017). Thus, the question whether the cation channels have an impact on reduced lipid accumulation and the signaling was addressed.

Since a concentration of 10 μM *trans-*pellitorine had no effect on gene expression of *PPARG* and *FAS* as well as the functional marker of lipid accumulation, the following experiments were carried out using 1 μM only. Thus, as a next step, the involvement of the TRPV1 and TRPA1 cation channels in the signaling pathway of *trans-*pellitorine was addressed by a concomitant application of the TRPV1 antagonist BCH or the TRPA1 antagonist AP-18 and 1 μM *trans-*pellitorine as effective concentration to differentiating 3T3-L1 cells. Effects of low doses of the vehicle ethanol as a TRPV1 agonist (Trevisani et al., 2002) on lipid accumulation in 3T3-L1 cells have been excluded in previous studies. The lipid lowering effect of 1 μM *trans-*pellitorine was diminished by addition of the TRPV1-inhibitor BCH, independent of an application during differentiation and maturation or maturation phase solely. In contrast, addition of the TRPA1-antagonist AP-18 diminished the effects of *trans-*pellitorine during differentiation and maturation, but not during maturation phase. On a molecular level, this effect was reproducible as well: downregulation of *PPARG* and *FAS* was diminished by the addition of BCH as well, whereas AP-18 application diminished the effects of *trans-*pellitorine on *PPARG*, but not on *FAS* expression. *PPARG* upregulation during adipogenesis of 3T3-L1 cells starts about 24–48 h after induction of differentiation using IBMX (Burton et al., 2004). We assume that TRPA1 antagonist AP-18 applied during this early to intermediate stages of adipogenesis may interfere with the downregulation of *PPARG* expression after *trans-*pellitorine treatment, which is also indicated by the gene expression data. In contrast, application of AP-18 starting 48 h after application of IBMX (maturation phase solely) seems not be sufficient to diminish the action of *trans-*pellitorine on PPARγ-levels and 24–48 h might be the critical time point for *trans-*pellitorine’s action on TRPA1. This hypothesis is also supported by the gene expression data, showing no effect of AP-18 *trans-*pellitorine-induced regulation of *FAS* expression, which is a marker of later stages of adipogenesis (Habinowski and Witters, 2001), but has to be investigated in detail in future studies. In addition, although there was no difference between an application during differentiation and maturation or during maturation phase alone at the higher test concentrations (0.1–10 μM), *trans-*pellitorine-treatment significantly reduced lipid accumulation also at the lower test concentrations of 1 and 10 nM when added during the early to intermediate stages as well. On the other hand, these data also indicate that TRPA1 might have other, so far unidentified targets which are critical in early, but not later stages of adipogenesis. This accounts also for the action of TRPV1, which might have other targets than *PPARG* and *FAS* in later stages of adipogenesis as well. Since the effects of *trans-*pellitorine could be diminished by inhibition of either TRPA1 or TRPV1 when added during differentiation, it is assumed that both ion channels or an interplay of TRPA1/TRPV1 is involved in the signaling of *trans-*pellitorine. Taken together, the results point to an involvement of the cation channels TRPV1 and TRPA1 in the signaling pathway of anti-adipogenic properties of *trans-*pellitorine, although *trans-*pellitorine does not directly activate TRPV1 or TRPA1 (Walker et al., 2017). We assume that *trans-*pellitorine might have modulating activity on TRPV1 and TRPA1, possibly via an interplay with other not yet identified molecular targets. In this context, it might be conceivable as well that TRPA1 and TRPV1 are affected via *trans-*pellitorine’s downstream-signaling. In addition, *trans-*pellitorine seems to address more than one signaling pathway to unfold its anti-adipogenic properties, which is also known for other phytochemcials like genistein (Zhang et al., 2009). Future studies will investigate whether *trans-*pellitorine is also effective in reducing body fat in *in vivo* studies, as it has been shown for the TRPV1 agonists nonivamide and capsaicin (Zhang et al., 2007; Hochkogler et al., 2017), but also for the TRPA1 agonist cinnamaldehyde (Tamura et al., 2012).

In summary, the present study demonstrates that application of low concentrations of *trans-*pellitorine inhibited lipid accumulation via affecting intermediate stages of adipogenesis in 3T3-L1 cells and short-term fatty acid uptake in 3T3-L1 cells. As a signaling pathway, we propose that *trans-*pellitorine has modulating activity on TRPV1 and TRPA1, which is leading to decreased levels of the pro-adipogenic factor PPARγ, possibly via the *miRNA let-7b*.

## Author Contributions

BL, VS, JH, JL, and GK designed the work and the single experiments and supervised the project. BL, MZ, and A-KH carried out the experiments and analyzed the data. BL performed statistical analyses and visualization of the data; BL, VS, JH, and JL interpreted the data. VS acquired funding for the work. BL wrote the original draft of the manuscript; VS, MZ, A-KH, JL, JH reviewed and edited the draft. All authors approved the final version of the manuscript.

## Conflict of Interest Statement

The co-authors JL, JH, and GK are employees of the Symrise AG. The other authors declare that the research was conducted in the absence of any commercial or financial relationships that could be construed as a potential conflict of interest.

## Footnotes

1 http://www.targetscan.org/mmu_61/Protein
